# Ethical factors determining ECMO allocation during the COVID-19 pandemic

**DOI:** 10.1186/s12910-021-00638-y

**Published:** 2021-06-01

**Authors:** Bernadine Dao, Julian Savulescu, Jacky Y. Suen, John F. Fraser, Dominic J. C. Wilkinson

**Affiliations:** 1 Oxford Uehiro Centre for Practical Ethics, Faculty of Philosophy, University of Oxford, Suite 8, Littlegate House, St Ebbes St, Oxford, OX1 1PT UK; 2 John Radcliffe Hospital, Oxford, UK; 3 Murdoch Children’s Research Institute, Melbourne, Australia; 4 Critical Care Research Group, The Prince Charles Hospital, Brisbane, Australia; 5 Faculty of Medicine, The University of Queensland, Brisbane, Australia

**Keywords:** Ethics, Ethical, ECMO, COVID-19, Resource allocation, Factors, Survey, Intensive care

## Abstract

**Background:**

ECMO is a particularly scarce resource during the COVID-19 pandemic. Its allocation involves ethical considerations that may be different to usual times. There is limited pre-pandemic literature on the ethical factors that ECMO physicians consider during ECMO allocation. During the pandemic, there has been relatively little professional guidance specifically relating to ethics and ECMO allocation; although there has been active ethical debate about allocation of other critical care resources. We report the results of a small international exploratory survey of ECMO clinicians’ views on different patient factors in ECMO decision-making prior to and during the COVID-19 pandemic. We then outline current ethical decision procedures and recommendations for rationing life-sustaining treatment during the COVID-19 pandemic, and examine the extent to which current guidelines for ECMO allocation (and reported practice) adhere to these ethical guidelines and recommendations.

**Methods:**

An online survey was performed with responses recorded between mid May and mid August 2020. Participants (n = 48) were sourced from the ECMOCard study group—an international group of experts (n = 120) taking part in a prospective international study of ECMO and intensive care for patients during the COVID-19 pandemic. The survey compared the extent to which certain ethical factors involved in ECMO resource allocation were considered prior to and during the pandemic.

**Results:**

When initiating ECMO during the pandemic, compared to usual times, participants reported giving more ethical weight to the benefit of ECMO to other patients not yet admitted as opposed to those already receiving ECMO, (*p* < 0.001). If a full unit were referred a good candidate for ECMO, participants were more likely during the pandemic to consider discontinuing ECMO from a current patient with low chance of survival (53% during pandemic vs. 33% prior *p* = 0.002). If the clinical team recommends that ECMO should cease, but family do not agree, the majority of participants indicated that they would continue treatment, both in usual circumstances (67%) and during the pandemic (56%).

**Conclusions:**

We found differences during the COVID-19 pandemic in prioritisation of several ethical factors in the context of ECMO allocation. The ethical principles prioritised by survey participants were largely consistent with ECMO allocation guidelines, current ethical decision procedures and recommendations for allocation of life-sustaining treatment during the COVID-19 pandemic.

**Supplementary Information:**

The online version contains supplementary material available at 10.1186/s12910-021-00638-y.

## Background

Extracorporeal membrane oxygenation (ECMO) is an advanced medical technique that provides support for patients with severe cardiac or respiratory failure, refractory to standard measures. ECMO has been used for patients suffering from COVID-19 with severe acute respiratory distress syndrome (ARDS), and/or haemodynamic compromise [1–5]. However, ECMO requires multi-disciplinary specialist care in an intensive care unit (ICU), and is resource-intensive even in ordinary times. It is a scarce resource during the COVID-19 pandemic, the allocation of which involves ethical considerations that may be different from usual times.

Prior to the pandemic, there have been several surveys of physician perspectives relating to ECMO [6–13], although few specifically focus on patient factors that influence ECMO allocation [6, 8, 12]. A 2019 study found that ECMO physicians making decisions about initiation of VV-ECMO for patients with severe respiratory failure, considered older patient age (46.9%), additional organ failures (37.7%), and prolonged mechanical ventilation (35.1%). Withdrawal of ECMO decisions were reported to be influenced by patient comorbidities (70.5%), patient’s wishes (56.0%), and etiology of respiratory failure (37.7%) [6]. Two surveys of ECMO directors on selection criteria for neonatal and paediatric patients, found significant variability in the selection criteria for ECMO in practice, although quality of life and neurological status were commonly used [8, 12].

During the pandemic, there has been limited international professional guidance regarding which ethical factors should be taken into consideration when allocating ECMO. Notably, the Extracorporeal Life Support Organization (ELSO) released a guidance document in May 2020 with general ethical recommendations for ECMO allocation during the COVID-19 pandemic (i.e. prioritise the young first, those with lowest co-morbidities first, healthcare workers first) [2]. There have also been numerous *clinical* interim guidelines released by the ELSO [2], the Society of Critical Care Medicine [14], and the World Health Organization (WHO) [1], among others [15]. These guidelines support referral for ECMO in adult and paediatric patients with ARDS from suspected COVID-19 who have refractory hypoxaemia despite lung protective ventilation strategy. However, they do not address ethical considerations or resource allocation.

There has also been active ethical debate about allocation of scarce critical care resources in general—(e.g. ventilators and intensive care beds)—during the COVID-19 pandemic. This literature has largely taken the form of ethical frameworks and recommendations published in medical journals [16–29], and ethical guidelines across different health organisations internationally [30–33]. For the most part, the recommendations arising from this literature have converged on the broad ethical principle of maximising medical benefit (conceived of as saving the most lives and life years)—which in practice calls for a consideration of factors such as patient age, comorbidities and functional status, which act as a proxy for their likelihood of recovery to an acceptable outcome so as to in turn reduce *overall* morbidity and mortality. Many have suggested that it would be ethical to withdraw treatment from one patient in favour of another patient with better prognosis, if doing so is necessary to maximise overall medical utility [16–19, 21, 23, 25, 26, 28, 32, 33], though this has been disputed [34]. Other common ethical themes have included allocation based on desert-based grounds (such as healthcare workers who contracted COVID-19 in the course of their work, or younger patients who have lived fewer years), patient autonomy, and equality of opportunity.

In this paper, we will examine first what factors appeared to influence ECMO decision-making in the first phase of the COVID-19 pandemic. We report the results of a small international exploratory survey of ECMO clinicians’ views on different patient factors in ECMO decision-making prior to and during the COVID-19 pandemic. We will then outline some current ethical algorithms and recommendations for rationing life-sustaining treatment during the COVID-19 pandemic, and examine the extent to which current guidelines for ECMO allocation (and reported practice) adhere to these ethical algorithms and recommendations.

## Survey

The aims of the survey were to assess what factors are considered for starting ECMO and for withdrawing ECMO during the COVID-19 pandemic; and whether these are any different from prior to the pandemic.

### Methods

An online survey (see Additional file 1: Appendix A for full survey) was created on Qualtrics—an online platform used to create and host surveys [35]. The survey included basic demographics, and information about the participants’ ECMO units, followed by four sections relating to the ethical factors involved in ECMO resource allocation in the units in which participants worked. The survey was based on an initial brief pilot survey that we had distributed to the same group of participants in April 2020 (Additional file 2: see Appendix B).

The survey included several questions designed to ascertain participants’ inclinations towards an egalitarian versus utilitarian approach to ECMO allocation in their units, with responses mapped onto a 7-point Likert scale; as well as their inclination towards an individualistic vs. collectivist approach to ECMO allocation before vs. during the COVID-19 pandemic, with responses mapped onto a 100-point scale.

Participants were asked to qualitatively indicate the frequency with which certain patient-related factors were considered when *initiating* ECMO, before versus during the pandemic. Participants were also asked their lower cutoff for patient probability of survival for initiating ECMO; their upper limit of age for initiating ECMO; whether they thought it would be ethical to give extra priority for ECMO to a healthcare worker who had contacted COVID-19 whilst working; whether their criteria for starting ECMO had changed during the COVID-19 pandemic; and whether the number of available beds for ECMO had changed during the COVID-19 pandemic. Participants were asked to indicate the extent to which they agree or disagree with several statements on why age is included in decisions about ECMO.

The survey concluded with a section about the patient factors involved in *withdrawing* ECMO, before vs. during the pandemic. Participants were asked the extent to which they consider the benefit to the individual being referred versus the potential benefit of ECMO to other patients. Participants were asked what probability of survival (%) with ECMO would be too low to continue ECMO, and the maximum number of days of ECMO they would consider reasonable, prior to vs. during the pandemic. Participants were asked whether they would consider discontinuing ECMO for a current patient (who has a low, but non-zero chance of survival) in order to provide ECMO to a good candidate referred patient. Finally, participants were asked if family do not agree with a recommendation to discontinue ECMO, if they would continue treatment, and if so for how many days, in usual circumstances versus during the pandemic.

### Participants

Participants were sourced primarily from the ECMOCard study group [36]. This is an international group of experts (at the time n = 120) taking part in a prospective international study of intensive care management of patients, including ECMO, during the early stages of the COVID-19 pandemic. Members of the ECMOCard group were emailed a link to the survey, and the study link was also distributed via a newsletter of ELSO. The survey was completed between May and August 2020.

Inclusion criteria for the survey included participants who work in a unit with ECMO, and who are aware of how decisions regarding ECMO are made in their unit. Some participants commenced but did not complete the survey. Responses that included at least questions relating to patient factors during and prior to the pandemic were analysed.

### Statistical analysis

Statistical analysis was conducted with IBM SPSS Statistics version 23 for Mac, using survey data imported as an SPSS file from Qualtrics. Descriptive and frequency statistics were used to describe the data. Paired samples t-tests were used to perform comparative analyses on responses across different questions. A *p* value of < 0.05 was considered statistically significant. Findings that achieved significance at only the *p* = 0.1 level were also reported, although the level of significance was always clarified with an accompanying statement.

## Results

### Participants

There were fifty-eight responses to the pilot survey. Forty-eight eligible responses to the formal survey were received (44 complete responses). Thirty-two of the participants were intensivists/ICU doctors, 9 were nurses, 2 were ECMO coordinators, 2 were cardiothoracic surgeons, 2 were perfusionists, and 1 was an administrator. Twenty-three (48%) had > 10 years of ECMO experience, 19 had 5–10 years of experience, and 6 had 1–5 years of experience. Survey responses were received from 19 different countries including 10 from the United States, 7 from Qatar, 6 from China, 6 from Singapore, 5 from Australia and a further 14 responses from 12 countries in Europe, Asia, South America and Africa.

### Unit characteristics and experience

Individual units were able to support a median of 8 ECMO patients at one time, (range 1–25). Thirty-seven units provided support for adult patients, 1 supported only paediatric/neonatal patients, and 10 units provided ECMO to both children and adults.

Forty-five participants reported that their ECMO unit had treated one or more patients with COVID-19. One of the 3 participants who had not had any COVID-19 patients, had been referred patients who were subsequently declined by their unit.

In a usual month (prior to the pandemic), 25 participants indicated that < 20% of their available maximum ECMO capacity was in use, 16 indicated 20–50%, five 50–80%, and two > 80%. In the past month (during the pandemic), 19 participants indicated < 20% of their available ECMO beds were occupied, 18 indicated 20–50%, 3 indicated 50–80%, and 8 indicated > 80%.

***Ethical principles.***

**Fig. 1:**
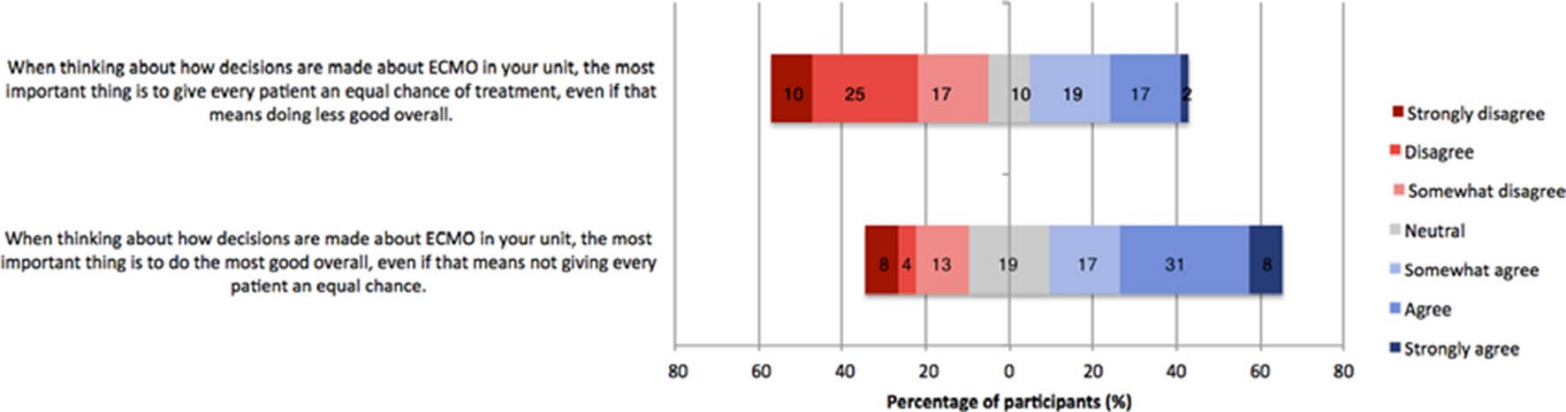
Agreement with ethical values relating to decisions about ECMO in their unit

Most survey participants agreed that the most important ethical principle for ECMO decision-making was doing the most good overall, a majority disagreed that it was most important to give every patient an equal chance of treatment (Fig. 1).

**Fig. 2:**
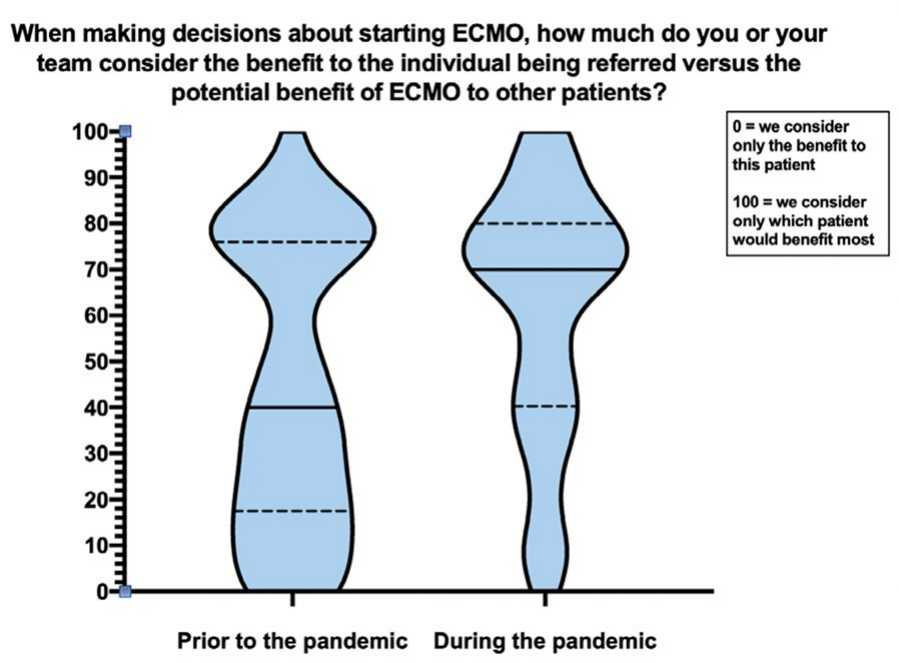
Consideration of the benefit to the individual patient being referred versus the potential benefit to other patients when making decisions about starting ECMO prior to versus during the pandemic

Prior to the pandemic, participants indicated that they tended to give more ethical weight to the benefit to individual patients, compared to the benefit to other patients (M = 44.63; SD = 31.25), when initiating ECMO. However, during the pandemic, participants were more likely to give more ethical weight to the benefit of ECMO to other patients, when initiating ECMO (M = 60.77; SD = 27.37), t(47) =  − 4.04, *p* < 0.001 (Fig. 2).

### Patient factors in decisions

**Fig. 3:**
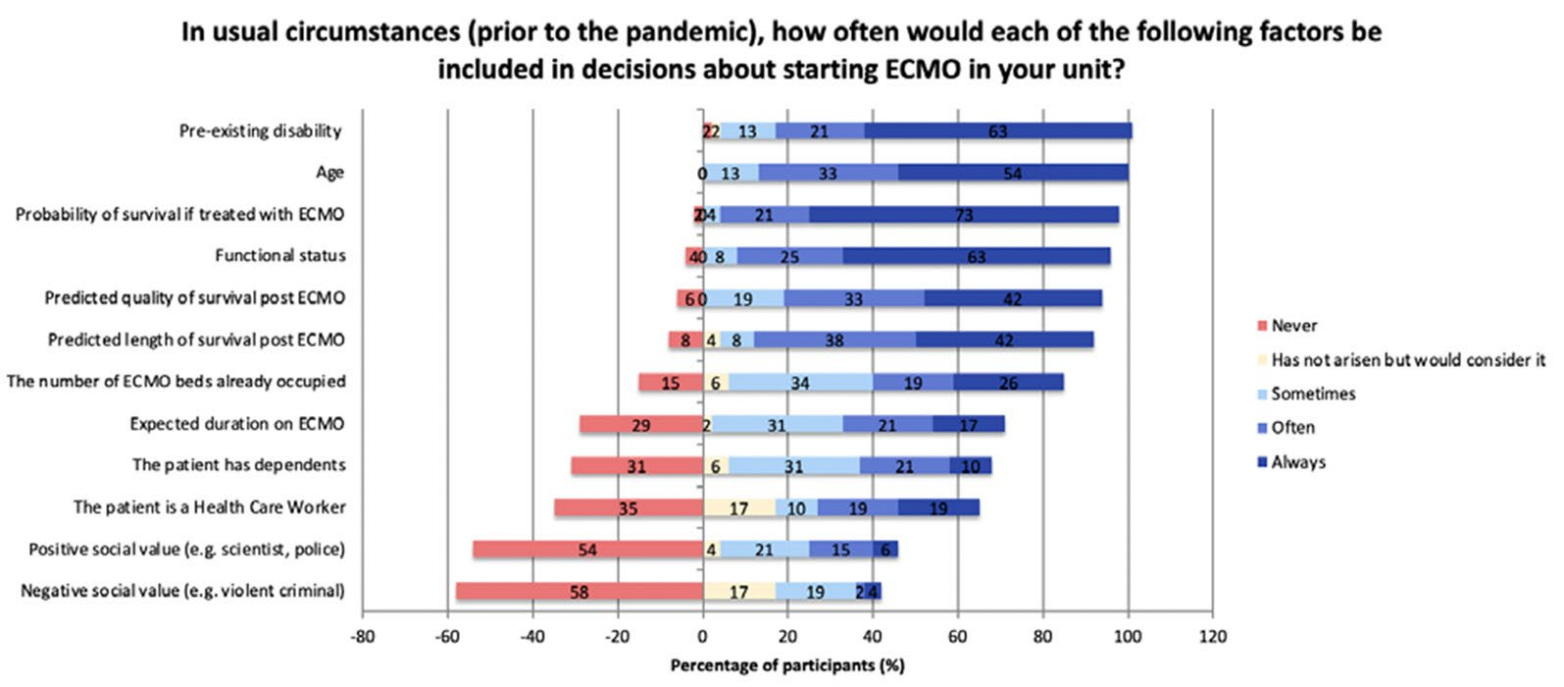
Frequency with which specified factors are included in decisions about starting ECMO in participants’ units, in usual circumstances (prior to the pandemic)

Four factors were cited by almost all participants as always or often being included in decisions about ECMO: Probability of survival if treated, Pre-existing disability, Functional status, and patient Age (Fig. 3). Predicted length and quality of survival post ECMO were always considered by 20 (42%) participants and often considered by a further 16–18 (33–38%) participants. The number of ECMO beds already occupied and the expected duration of ECMO treatment was considered at least sometimes by 79% and 71% of participants respectively. In contrast, a majority of participants reported never considering the patient’s positive or negative social value in decisions (54–58%). There were similar responses in the pilot survey (Additional file 2: Appendix B).

Participants gave a median survival threshold (a probability of survival below which ECMO would not be started), of 25% (M = 29.77%; IQR = 18%).

Forty of 48 participants reported an upper age limit for ECMO in usual circumstances, with a median of 65 years (range 60–80; 65 was also the most common response, cited by 16 participants).

**Fig. 4:**
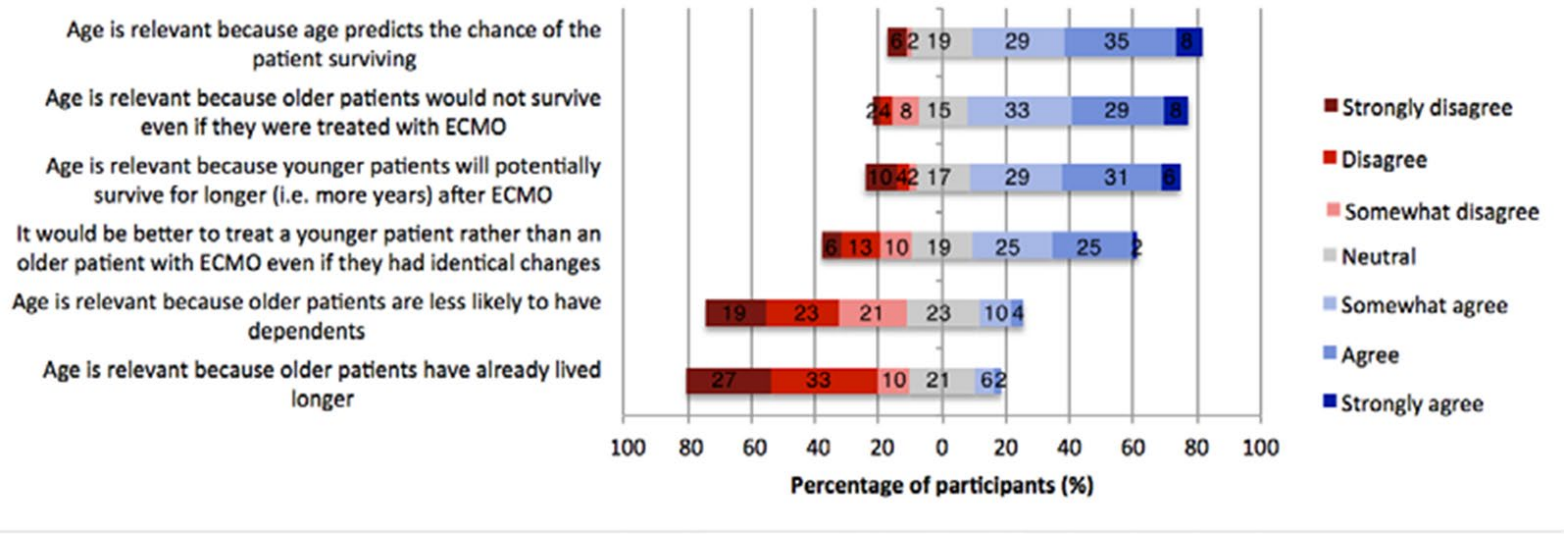
Responses for extent to which participants agree or disagree with several statements relating to why age is included in decisions about ECMO

When asked about why age is relevant to decisions, most participants cited chance of survival, agreeing that older patients would not survive or would have a lower chance of survival than younger patients (Fig. 4). The majority of participants also indicated that age is relevant for decisions because a younger patient will potentially survive for longer. They did not generally endorse the idea that age should be considered because an older patient has already had more years of life.

### Decisions starting ECMO—pandemic

Twenty-nine of 45 participants stated that their criteria for starting ECMO had changed during the COVID-19 pandemic. Those respondents cited lower age limits, decreased ECMO machine availability, and stricter inclusion criteria.

Forty of 45 participants reported an upper age limit for ECMO during the pandemic, with a median of 65 years (range 50–80; 65 was also the most common response, cited by 15 participants).

Seventeen of 44 participants indicated that the number of available beds for ECMO had changed during the COVID-19 pandemic; almost all of these indicated that ECMO capacity increased.

**Fig. 5:**
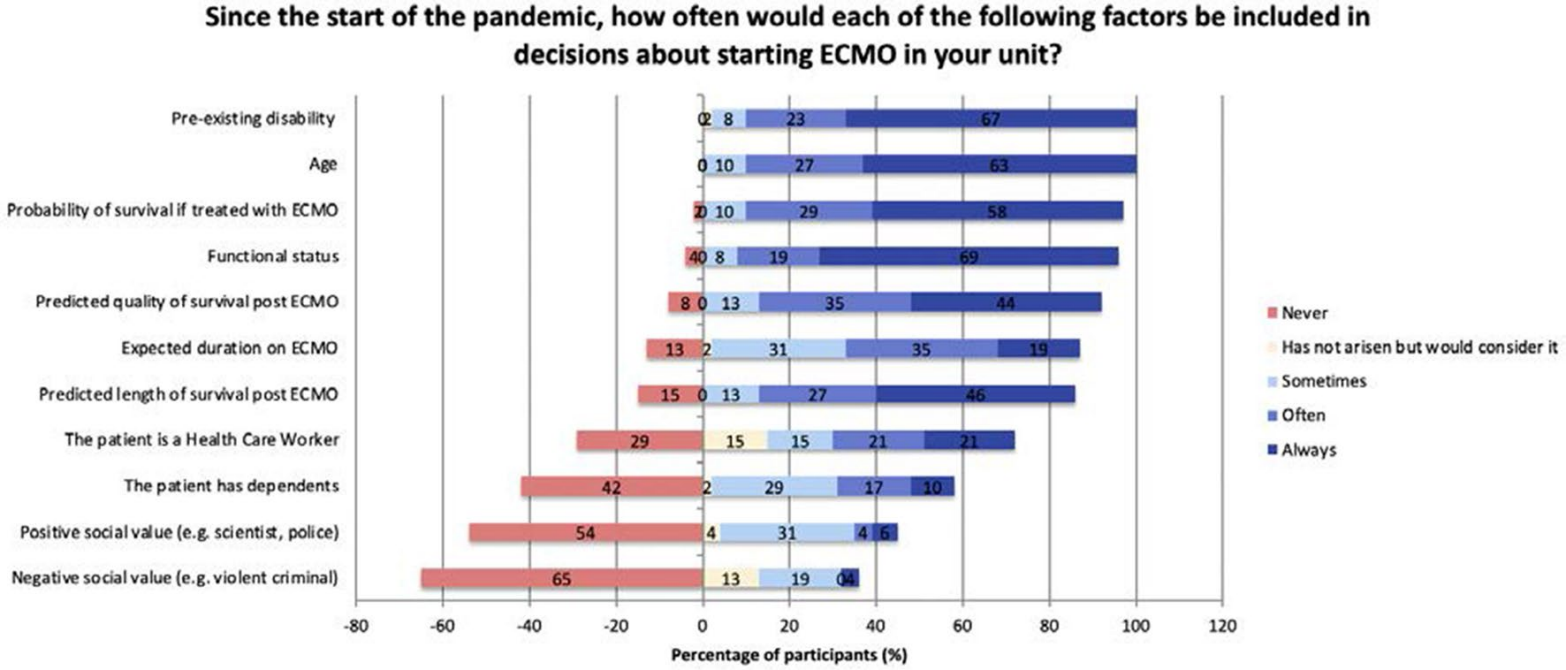
Frequency with which specified factors are included in decisions about starting ECMO in participants’ units, since the start of the pandemic

Participants were somewhat more likely to report that expected duration of ECMO was always or often considered during the pandemic compared to prior to the pandemic (54% vs. 38%) (M = 2.44, SD = 1.009; M = 2.79, SD = 1.110), t(47) = 3.13, *p* = 0.003 (Fig. 5). Participants were slightly less likely to report that probability of survival was always or often considered during the pandemic (88% vs. 94%) (M = 1.56, SD = 0.769; M = 1.35, SD = 0.668), t(47) =  − 2.87, *p* = 0.006. None of the other factors assessed reached statistical significance at the *p* = 0.05 level.

Thirty-four participants (71%) indicated that since the start of the pandemic, they have—or would theoretically—consider whether the patient is a healthcare worker, when making decisions about starting ECMO in their units. Of these, 81% agreed that it would be ethical to give extra priority to a healthcare worker who had contacted COVID-19 whilst working. Furthermore, 21 participants stated that they would consider treating a healthcare worker with COVID-19 above their usual age limit, while the remaining 11 participants stated that they would not.

### Decisions about stopping ECMO

**Fig. 6:**
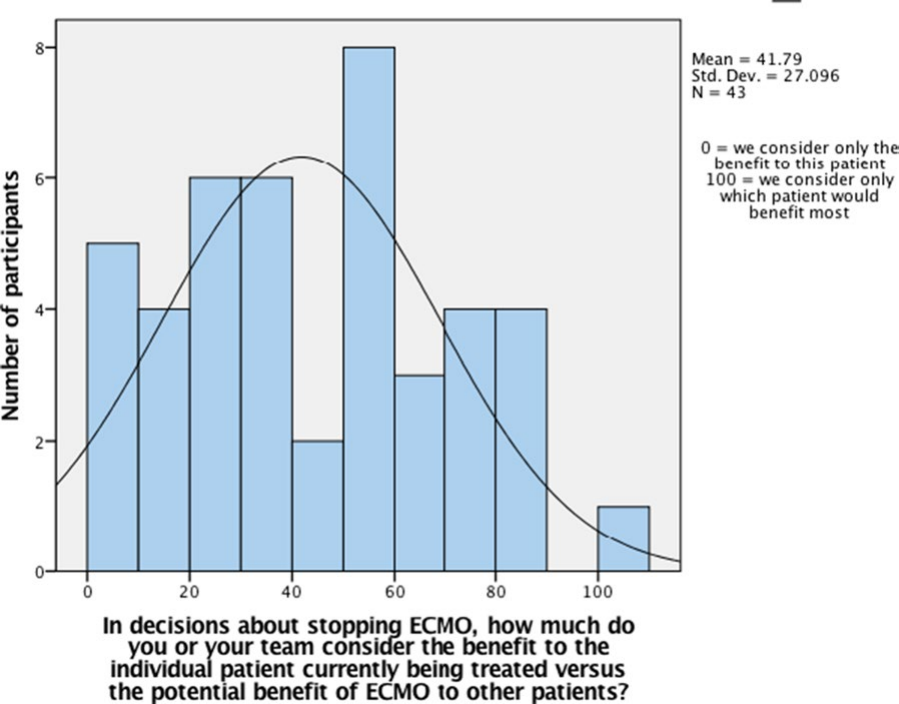
Responses for extent to which participants ‘consider the benefit to the individual being referred versus the potential benefit of ECMO to other patients’ when making decisions about stopping ECMO

In decisions about stopping ECMO, participants reported giving most consideration to the benefit to the individual patient (rather than considering which patient would benefit most) (M = 41.79, SD = 27.10) (Fig. 6).

**Fig. 7:**
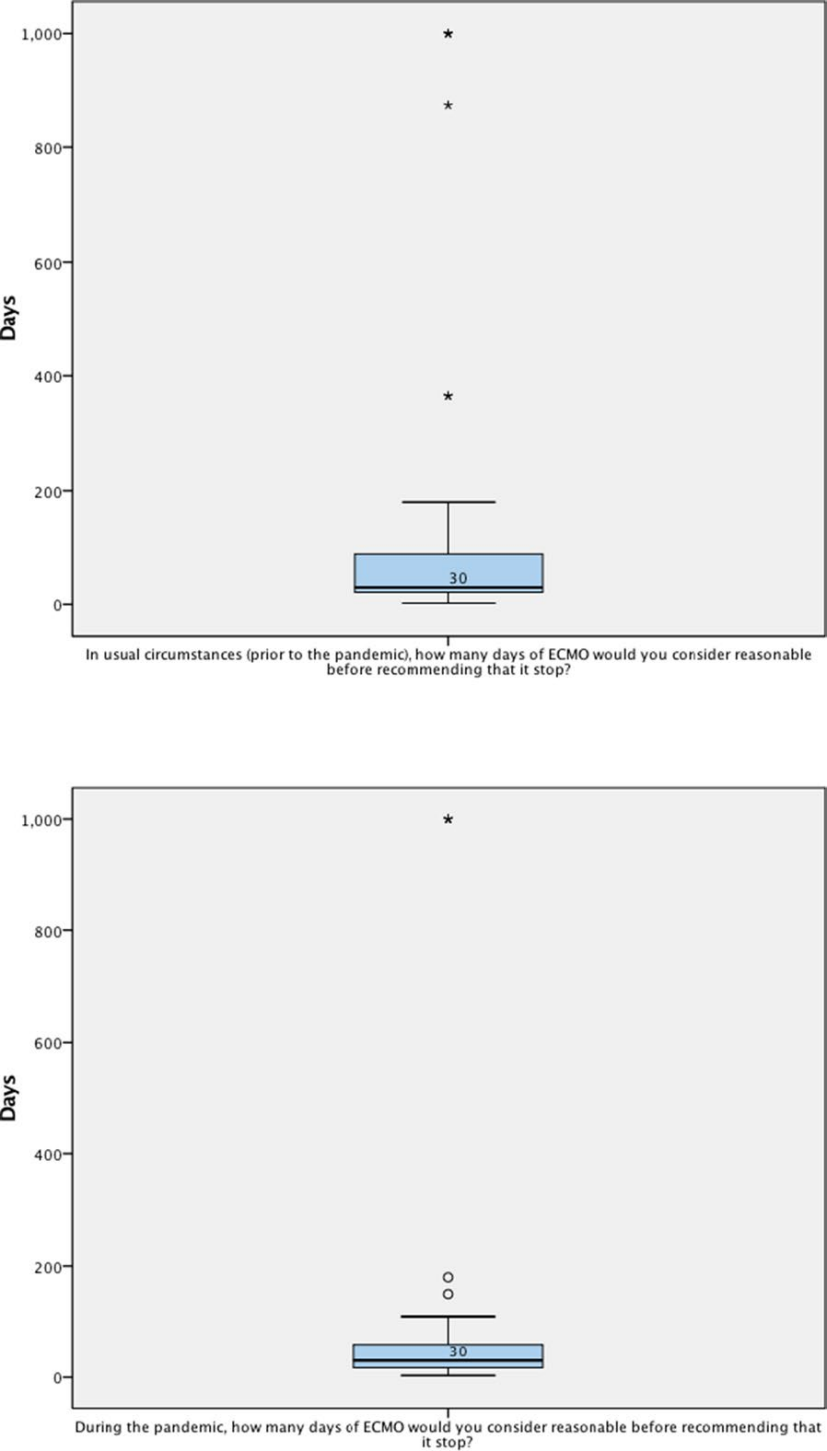
Days of ECMO considered reasonable before recommending that it stop, in usual circumstances (prior to the pandemic) versus during the pandemic

**Fig. 8:**
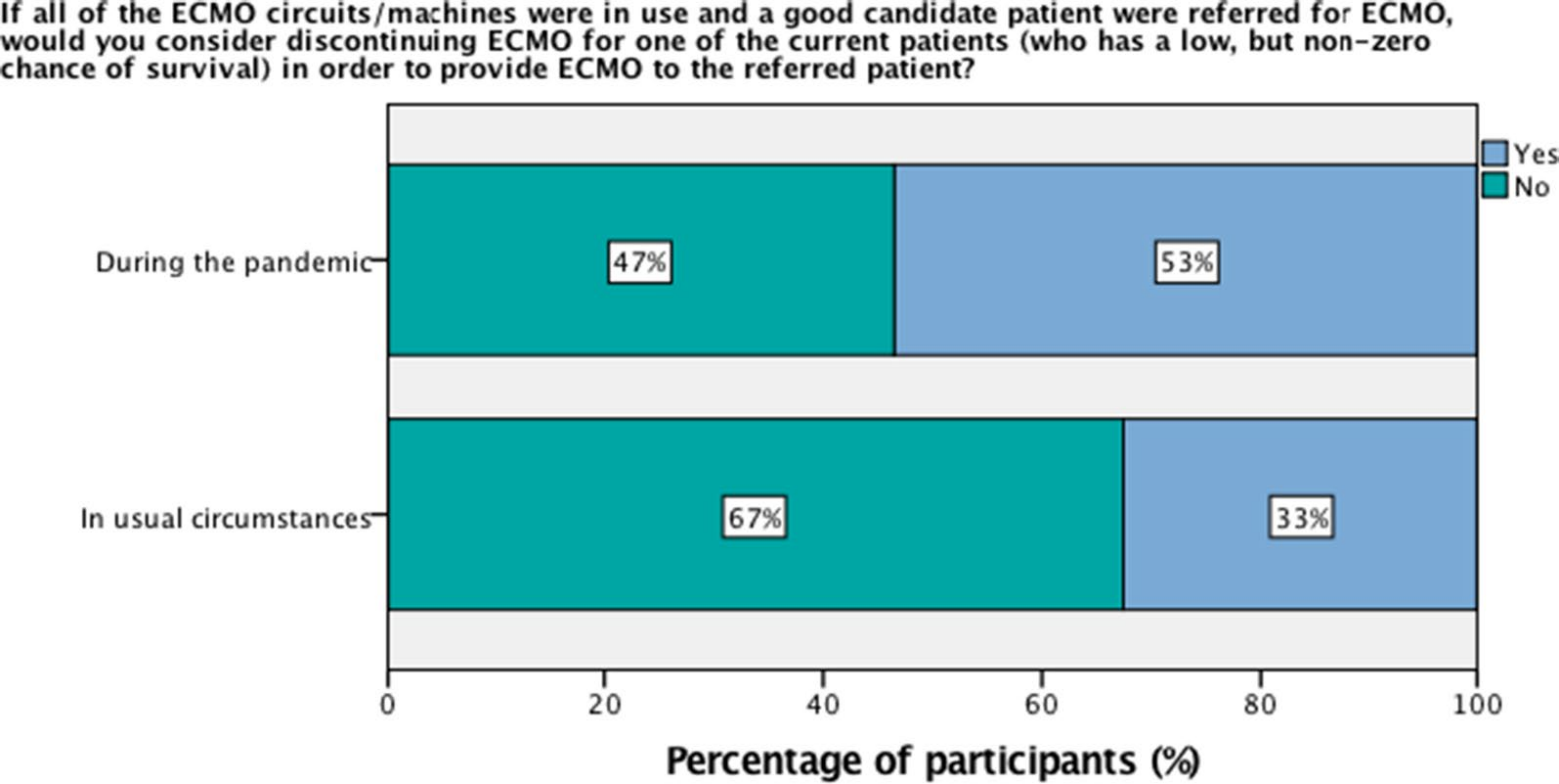
Percentage of participants that would consider discontinuing ECMO for one of the current patients (who has a low, but non-zero change of survival) in order to provide ECMO to the referred patient, if all of the ECMO circuits/machines were in use and a good candidate patient were referred for ECMO, in usual circumstances versus during the pandemic

Participants reported a median survival threshold for discontinuing ECMO of 20% (below this chance, ECMO would potentially not be continued) (M = 21%; IQR 20%). The maximum duration of ECMO considered reasonable was similar prior to and during the pandemic, at a median of 30 days in both contexts (IQR = 69, M = 145.26, SD = 278.27 prior to pandemic; IQR = 46, M = 109.42, SD = 249.70 during pandemic) (Fig. 7). However, participants were significantly more likely during the pandemic (M = 1.47, SD = 0.505) when compared to prior to the pandemic (M = 1.67, SD = 0.474), t(42) = 3.33, *p* = 0.002 to consider discontinuing ECMO from a current patient with low chance of survival if all of the ECMO circuits/machines were in use and a good candidate patient were referred for ECMO (53% during pandemic vs. 33% prior) (Fig. 8).

**Fig. 9:**
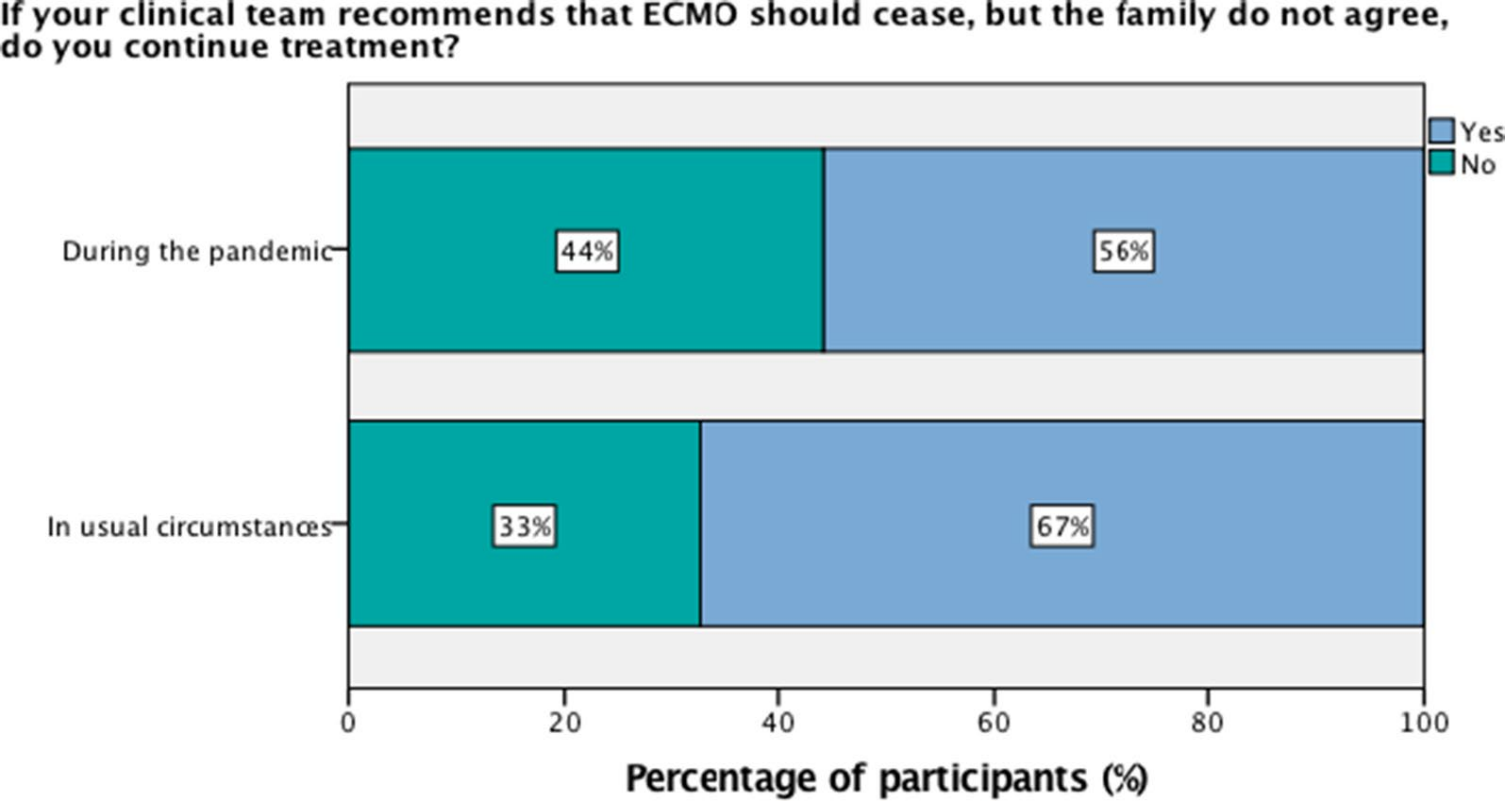
Percentage of participants that would continue treatment, if their clinical team recommends that ECMO should cease, but family do not agree, in usual circumstances versus during the pandemic

If the clinical team recommends that ECMO should cease, but family do not agree, the majority of participants indicated that they would continue treatment, both in usual circumstances (67%) and during the pandemic (56%); participants were a little less likely to continue treatment during the pandemic (M = 1.44, SD = 0.502) when compared to in usual circumstances (M = 1.33, SD = 0.474), t(42) =  − 1.703, *p* = 0.096, though this was not statistically significant (Fig. 9). When asked for how many days they would continue treatment, the participants who responded ‘yes’ to continuing treatment largely cited ‘a few days (2–3)’, ‘7 days’, ‘until the family accepts’, and ‘until ethics approval’.

## Discussion

### Principal findings

This international survey explored the experience and views of ECMO practitioners during the pandemic from 17 different countries. The survey assessed which factors would be included in decisions, the potential ethical justification for different decisions, and any differences in practice associated with the special circumstances of the pandemic.

In general, most of those taking part in the survey reported that when thinking about how decisions are made about ECMO in their unit, the most important thing is to do the *most good overall,* even if that means not giving every patient an equal chance. Factors relevant to maximising the benefit of ECMO treatment were frequently considered, including chance of survival, duration of survival, age and quality of life.

Most participants stated that their criteria for starting ECMO had changed during the COVID-19 pandemic, with lower age limits, decreased ECMO machine availability, and stricter inclusion criteria.

In the context of the pandemic, participants were more likely to consider the potential benefit of ECMO to other patients. Interestingly, most indicated that since the start of the pandemic, they have—or would—consider whether the patient is a healthcare worker. A majority agreed that it would be ethical to give extra priority for ECMO to a healthcare worker who had contacted COVID-19 whilst working.

Decisions about stopping ECMO were generally focused on the benefit of treatment to the individual patient rather than to other patients needing it. However, during the pandemic more participants were willing to consider discontinuing ECMO, from a current patient who has a low, but non-zero chance of survival, in order to provide ECMO to a good candidate patient referred for ECMO. Global differences in legal frameworks underpinning healthcare infrastructure (e.g. in the US) may have affected willingness to discontinue ECMO, however we had insufficient data to extract meaningful conclusions from stratifying responses by geographic region. We were unable to assess how decision-making might have been affected by other changes related to the pandemic (for example, difficulty in arranging face-to-face meetings with families).

During the pandemic, the vast majority of participants in our study who noted any change in the number of available ECMO beds indicated that it had actually increased. Participants also reported that a higher proportion of their available ECMO beds were occupied during the pandemic. It should be noted that provision of ECMO entails a resource demand that is typically higher than that of mechanical ventilators—particularly with respect to ICU staffing. When demand for ICU staffing surpasses available resources, limitation or cessation of ECMO provision may result in more non-ECMO patients being treated [37], and Supady et al. [38] argue that a decision to curtail or continue ECMO in this context should be ethically deliberate and consistently applied with use of triage guidelines. Although we did not further characterise the change in ECMO bed availability in participants’ units as it was outside the scope of our study, it is an ethically important topic that would benefit from further research.

Most participants indicated that they would continue ECMO treatment if it was no longer clinically indicated, but family did not agree with withdrawing ECMO, in both usual circumstances and during the pandemic. Notably, the ethico-legal factors underpinning this scenario were recently demonstrated in the Court of Protection (UK) in a case where a patient with COVID-related severe pulmonary fibrosis, pulmonary embolisms and cardiac arrest was placed on ECMO for 15 weeks at the wishes of his family and despite strong recommendations from the medical treating team that further ECMO treatment was futile and constituted mere prolongation of pain and distress to the patient [39]. Our survey did not attempt to further delineate the reasons that participants would continue ECMO if it were not clinically indicated but family wished to continue ECMO.

### Limitations

This survey was hampered by a relatively low response rate (potentially influenced by high clinical workload in intensive care units during the pandemic). Because of the small sample size (n = 48), it is not possible to generalise about the experience or practice of ECMO professionals in normal times or during the COVID-19 pandemic. Decision-making may have changed over the course of the pandemic (including as more information was known about the course of COVID-19). However, the wide international background of participants potentially provides some insights into global ECMO allocation decisions and indicates some potential hypotheses about how clinicians approach decisions. We had similar responses in the short pilot survey with a slightly higher response rate (n = 58) (Additional file 2: Appendix B).

We will use the results of this survey as a starting point for a discussion of the ethical factors that are and/or should be relevant when making decisions about allocation of resources in acutely severe periods of shortage.

### Ethical discussion

One crucial ethical consideration, during a pandemic, is the need to allocate scarce medical treatment. ECMO is a paradigm scarce resource, and is potentially rationed even in ordinary times, provided only to some patients with refractory respiratory or circulatory failure with pre-determined criteria based on likelihood of survival. During the COVID pandemic, resource allocation becomes even more relevant to ECMO decision-making because of the increased number of patients with respiratory failure and the shortage of intensive care capacity at some time points.

There are two types of guidance that ECMO practitioners might draw on to inform their decision-making in the difficult circumstances of the pandemic. Professional guidance from national or international organisations might provide updated clinical criteria for allocating treatment. Alternatively, or additionally, guidance from medical ethicists might be used as the ethical basis for locally developed policy or procedures.

We will compare some key features of pandemic-specific ECMO allocation guidelines with the conclusions of two papers published during the first wave of the pandemic [16, 18] and our survey participants’ responses.

#### Professional guidance

The international organisation ELSO (Extracorporeal Life Support Organization) released a guidance document in May 2020 with general recommendations for ethical considerations in ECMO allocation during the COVID-19 pandemic. The guidance emphasised that ECMO had a potential role in the treatment of patients with COVID-19 when conventional management has failed. It may not be able to be provided if hospital resources are overwhelmed and required for other patients. According to the guidance, when resources are limited, the youngest patients with low or no co-morbidities should be the highest priority, while health care workers are also a high priority. Age “should be considered” in allocation because of its relationship with prognosis [2]. Patients with “Severe central nervous damage” or “significant co-morbidities” were suggested to be excluded. While the document discusses termination of ECMO treatment that is futile (e.g. after 21 days), there is no discussion of reallocation of treatment in a situation where newly arriving patients need treatment and cannot be accommodated.

#### Ethical guidance

The ethical principles underlying the ELSO guidance were not explicit. Early in the course of the pandemic, an influential paper in the New England journal of medicine by Emanuel et al. outlined six recommendations for allocating medical resources in the COVID-19 pandemic [16]. Relevant to ECMO decision-making these included *maximising benefits* (i.e. saving more lives and more years of life, even if that means removing a patient from a ventilator or an ICU bed to provide it to others in need); prioritising *healthcare workers* (for their instrumental value in the pandemic response); avoiding allocating on a first-come, first-served basis; and applying the same principles to all COVID-19 and non-COVID-19 patients.

We (JS and DW) have published an algorithm for rationing life-sustaining treatment (such as ECMO, ventilators, ICU, and organs) during the COVID-19 pandemic, using what we took to be several essential ethical principles that ought to guide resource allocation in any country or setting, with optional ethical elements that would vary depending on the weight given to certain ethical values across different countries [18]. The relevant principles included upholding *patient autonomy* in the context of competently requesting or refusing treatment; *‘equal treatment for equal need’* e.g. ‘first come first served’ if sufficient resources are available; and foregoing ‘first come first served’ for ‘*save the most lives’* (i.e. *utilitarianism, and/or contractualism*), in the interests of ‘*temporal neutrality’*, if insufficient resources are available. The latter explicitly endorsed the principle of reallocating treatment if required.

In the event of insufficient resources despite following the above principles, optional ethical principles to be considered include ‘*lottery’* (or ‘first come first served’); ‘*predicted length and quality of life’* e.g. quality-adjusted life-years (QALYs); ‘priority’ on either *utilitarian* or *desert-based* grounds (e.g. younger patients having lived less years, or healthcare workers who contracted COVID-19 at work); and ‘trial of treatment’ in the spirit of ‘*equality of opportunity’*.

#### Comparison

Table 1 compares the professional and ethical guidance with the evidence from our survey of factors that may be considered internationally by ECMO professionals. Here we highlight three interesting areas of overlap and difference.

**Table 1:** Comparison between survey participants’ responses, current ECMO allocation guidelines (ELSO), and current ethical algorithms and recommendations for rationing life-sustaining treatment during the COVID-19 pandemic

Ethical factors prioritised in ECMO allocation during COVID-19 pandemic (i.e. resource scarcity)							
	Save the most lives	Younger patients	Equality of opportunity	Healthcare workers	Do *not* allocate on first-come-first-served basis	Participants of COVID-19 clinical trials	Reallocate ECMO if required to maximise overall benefit
Survey participants	✓	✓ Older patients less likely to survive ECMO	X	✓	X	N/A	X Largely neutral
ECMO allocation guidelines (ELSO)	✓ Lowest co-morbidities	✓	X	✓	X	N/A	N/A
Ethical algorithm (Savulescu et al.)	✓ Predicted length and quality of life (QALY)	✓ Save the most lives; and desert-based	✓	✓ Desert-based (i.e. contracted COVID-19 at work)	✓ Temporal neutrality	N/A	✓
Ethical recommendations (Emanuel et al.)	✓ Save more lives, *and* more years of life	✓ Prioritise young, severely ill patients (more likely to recover with treatment)	✓ Apply the same principles to COVID-19 and non-COVID-19 patients	✓ Desert-based (for ventilators), and instrumental value (for testing/PPE/ICU beds)	✓	✓ Instrumental value; and desert-based	✓

The three guidelines would support prioritisation of younger patients. In the ELSO guidance, this is linked to prognosis (i.e. chance of survival); however, in the ethical guidelines it is related to both chance and duration of survival (number of life years saved) [16, 18]. All of the participants to our survey indicated that age was included in ECMO decision-making. Many units used an age cut-off for ECMO, and some indicated that this had been lowered in the setting of the pandemic. This is clearly a highly important, though controversial factor in the setting of COVID-19. The greatest morbidity and mortality from the pandemic has been in older patients. However, there has been ethical controversy about suggestions of rationing intensive care treatment on the basis of age, and proposals to do so in Italy at the height of the initial wave of the pandemic attracted considerable ethical criticism [40, 41]. One important question is whether simple age-based cut-offs can be justified for ECMO allocation. While such cut-offs are procedurally simple to apply, they are inevitably arbitrary and would potentially discriminate against some older patients who may be physically fit and have better prognosis than other younger patients. It would be better for decisions to be explicitly linked to predictions of probability and duration of survival.

Participants in our survey were divided about discontinuation of ECMO treatment in order to provide treatment to another patient with better prognosis (a higher proportion supported this in the setting of the pandemic). Interestingly, this was not mentioned in the ELSO pandemic guidance. However, it does feature in ethical recommendations. Both Savulescu et al. and Emanuel et al. argue that a ‘first come first served approach’ to life-sustaining medical treatment is ethically flawed, and that consideration should be given to *re-allocation* of treatment if necessary [34]. The ELSO guidance indicates that treatment may be regarded as futile and discontinued after 21 days. However, it is clear that longer runs of ECMO can in some circumstances lead to survival and are not obviously futile. The key ethical reason to avoid such long treatment runs is that this would potentially mean that fewer patients can benefit from ECMO. In the setting of a pandemic, it would be justified to recommend much shorter cut-off lengths for discontinuing ECMO. Professional guidelines should consider recommending reduced duration trials of treatment during a pandemic.

Finally, all sources of guidance indicated potential support for giving priority to healthcare workers. This is highly relevant for COVID-19 because of the relatively large number of healthcare workers who have developed severe life-threatening illness as a consequence of exposure to the virus in the course of their work [42–44]. It appears that exposure to high viral load (particularly in association with aerosol generating procedures) may be a risk factor for more severe illness [45] Our survey participants were somewhat more divided about consideration of this factor—perhaps because it is a departure from normal practice. In ordinary circumstances, ethical guidelines would not direct special treatment for health professionals who become unwell—that would be potentially regarded as unjust favouritism by the wider community. However, the specific circumstances of the pandemic may change that. The ethical argument in favour of prioritising healthcare workers is not typically utilitarian. (It is not that such patients have a better prognosis, and unlikely those who are sick enough to need ECMO will be able to return to the front line soon). Rather, it is based on a notion of desert and (perhaps) compensation for the sacrifices/risks borne by such workers. However, no existing guidance indicates what degree of priority for healthcare workers would be justified. If there are multiple patients competing for ECMO treatment, and a healthcare worker with worse prognosis is prioritised, that may reduce overall numbers of patients surviving. To avoid an undue effect on patient access to treatment, if ECMO is commenced for a healthcare worker with severe COVID-19, that should potentially be discontinued on the same basis and at the same time point as for other patients.

## Conclusion

During the COVID-19 pandemic, intense pressure on existing resources has impacted on delivery of the highly specialised treatment ECMO. Our survey suggests that this pressure may have affected practice in some centres. Most salient of these were greater consideration of the potential benefit of ECMO to other patients; consideration of priority to healthcare workers; more willingness to consider reallocation of ECMO; and less willingness to continue treatment during the pandemic if a family do not agree with recommendations to cease ECMO.

A comparison of the ethical factors prioritised by practitioners, with ECMO allocation guidelines, and current ethical algorithms and recommendations, found some overlap, but also some differences. Ethical guidance for ECMO in subsequent waves of the pandemic should clarify recommendations on age/survival duration, reallocation of treatment/treatment trials, and the relative priority of healthcare workers.

## Supplementary Information

**Additional file 1.** Appendix A: Survey.

**Additional file 2.** Appendix B: Pilot survey.

## Data Availability

All data analysed during this study are included in this published article and its supplementary information files.

## Abbreviations

ARDS: Acute respiratory distress syndrome
COVID-19: Coronavirus disease 2019
ECMO: Extracorporeal membrane oxygenation
ECMOCard: ECMO coronavirus acute respiratory disease
ELSO: Extracorporeal life support organization
ICU: Intensive care unit
VV-ECMO: Veno-venous extracorporeal membrane oxygenation
WHO: World Health Organization
