# Supplementary figures and images for: Ethical factors determining ECMO allocation during the COVID-19 pandemic
Source: BMC Med Ethics. 2021 Jun 1;22:70. doi: 10.1186/s12910-021-00638-y (PMC8169422; doi:10.1186/s12910-021-00638-y)

**APPENDICES**

**Appendix A: Survey**

**See attached document “Survey”**

**Appendix B: Pilot survey**

*
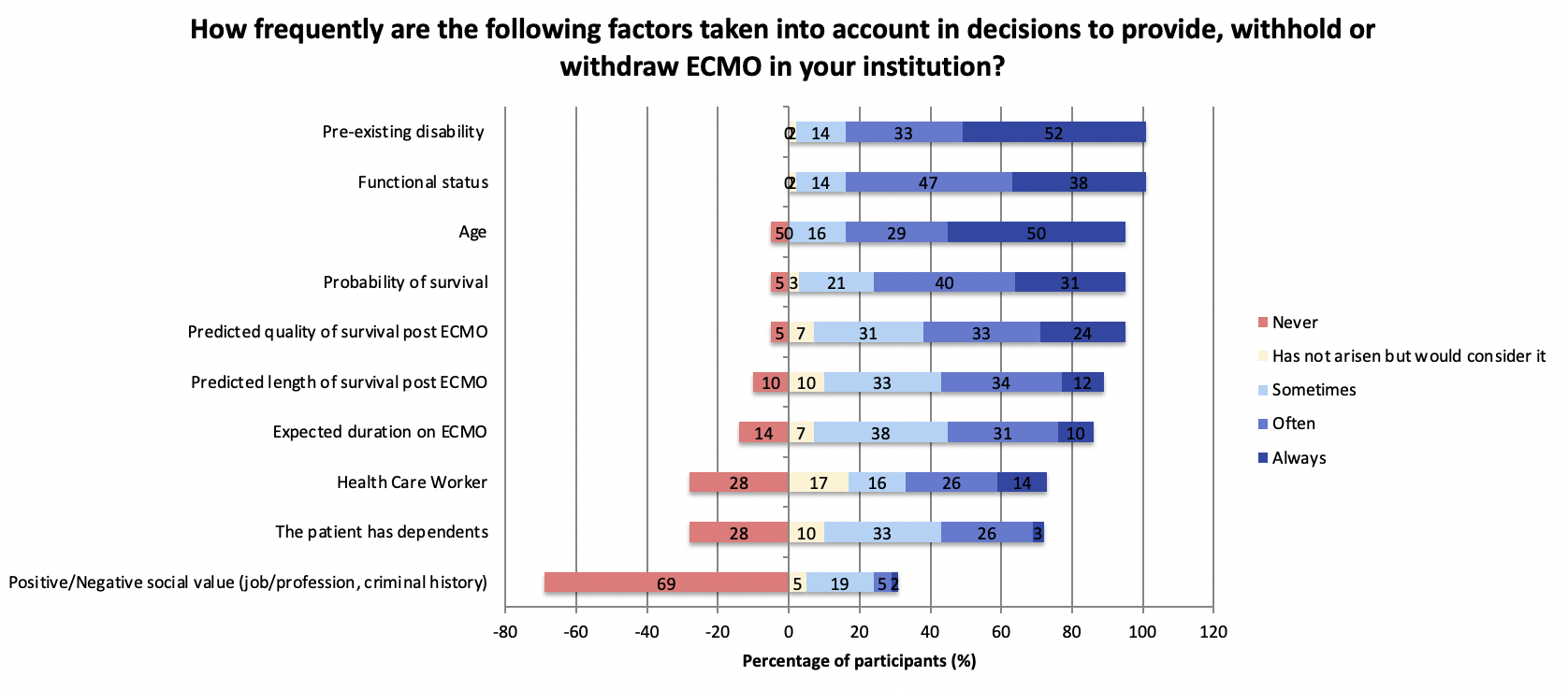
*

Supplement: Supplementary file 1 — Additional file 1. Appendix A: Survey. [file 12910_2021_638_MOESM1_ESM.docx]
