# Supplementary material for: Ethical factors determining ECMO allocation during the COVID-19 pandemic
Source: BMC Med Ethics. 2021 Jun 1;22:70. doi: 10.1186/s12910-021-00638-y (PMC8169422; doi:10.1186/s12910-021-00638-y)
Supplement: Supplementary file 2 — Additional file 2. Appendix B: Pilot survey. [file 12910_2021_638_MOESM2_ESM.pdf]

# Ethical factors determining ECMO allocation prior to and during the COVID-19 pandemic

Start of Block: Preamble

Q0 ***General Information***The aims of this study are to assess what patient characteristics are considered for starting ECMO and what factors are considered for withdrawing ECMO during the COVID-19 pandemic; and whether these are any different from prior to the pandemic. We appreciate your interest in participating in this online survey. You have been invited to participate as you are a member of the ECMOCard study group. Please read through this information carefully before agreeing to participate by ticking the 'yes' box below. You may ask any questions before deciding to take part by contacting the researcher (details below). We (Professor Wilkinson and Professor Savulescu) at the University of Oxford in collaboration with Dr Bernadine Dao, Dr Jacky Suen and Prof John Fraser are investigating ethical issues relating to the pandemic. You will be given some questions relating to factors determining access to ECMO at your intensive care unit, both during and prior to the COVID-19 pandemic. It should take about 15 minutes. ***Do I have to take part?***Please note that your participation is voluntary. If you do decide to take part, you may withdraw at any point during the questionnaire for any reason before submitting your answers by pressing the 'Exit' button/closing the browser. ***How will my data be used?***Your answers will be completely anonymous, and we will take all reasonable measures to ensure that they remain confidential.Individual intensive care units will not be identified. In the event that only a small number of units are reported from an individual country, country level responses for that particular country will not be reported, so as to maintain de-identification of individual units.Your data will be stored in a password-protected file and may be used in academic publications. Your IP address will not be stored. Research data will be stored for a minimum of three years after publication or public release.The data that we collect from you may be transferred to, stored and/or processed at a destination outside the UK and the European Economic Area ("EEA"). By submitting your personal data, you agree to this transfer, storing or processing. ***Who will have access to my data?***Qualtrics is the data controller with respect to your personal data and, as such, will determine how your personal data is used.  Please see their privacy notice here [https://www.qualtrics.com/privacy-statement/].  Qualtrics will share only fully anonymised data with the University of Oxford, for the purposes of research.Your responses may be shared with our aforementioned collaborators.Responsible members of the University of Oxford and funders may be given access to data for monitoring and/or audit of the study to ensure we are complying with guidelines, or as otherwise required by law. The Principal Researcher is Professor Dominic Wilkinson, who is attached to the Oxford Uehiro Centre for Practical Ethics at the University of Oxford.This project has been reviewed by, and received ethics clearance through, the University

of Oxford Central University Research Ethics Committee [R69730/RE001]. ***Who do I contact if I have a concern about the study or I wish to complain?***If you have a concern about any aspect of this study, please speak to Professor Dominic Wilkinson (Dominic.wilkinson@philosophy.ox.ac.uk), and we will do our best to answer your query. I will acknowledge your concern within 10 working days and give you an indication of how it will be dealt with.  If you remain unhappy or wish to make a formal complaint, please contact the Chair of the Research Ethics Committee at the University of Oxford who will seek to resolve the matter as soon as possible: Chair, Social Sciences & Humanities Interdivisional Research Ethics Committee; Email: ethics@socsci.ox.ac.uk; Address: Research Services, University of Oxford, Wellington Square, Oxford OX1 2JD
 Please note that you may only participate in this survey if you are at least 18 years of age. If you agree to participate and have read the terms above, please check the relevant box below to get started.

- ○ Yes, I agree to take part  (1)
- ○ No, I do not wish to take part  (2)

*Skip To: End of Survey If General Information The aims of this study are to assess what patient characteristics are conside... = No, I do not wish to take part*

**End of Block: Preamble**

---

**Start of Block: Inclusion**

Q1 Do you work in a unit that provides ECMO?

- ○ Yes  (1)
- ○ No  (2)

*Skip To: End of Survey If Do you work in a unit that provides ECMO?  = No*

Q2 Are you aware of how decisions are made in your unit relating to starting and stopping ECMO?

- ○ Yes  (1)
- ○ No  (2)

*Skip To: End of Survey If Are you aware of how decisions are made in your unit relating to starting and stopping ECMO? = No*

End of Block: Inclusion

Start of Block: Demographics

Q3 What is your profession?

- ○ Intensivist/ICU doctor (1)
- ○ Perfusionist (2)
- ○ Nurse (3)
- ○ Administrator (4)
- ○ Other (please specify) (5) ________________________________________________

Q4 How many years of relevant experience do you have?

- ○ <1 year (1)
- ○ 1-5 years (2)
- ○ 5-10 years (3)
- ○ >10 years (4)

Q5 In which country is your unit based?

▼ Afghanistan (1) ... Zimbabwe (196)

End of Block: Demographics

Start of Block: Unit

Q6 What is the maximum number of patients that your unit is able to support on ECMO at the same time?

▼ 1 (1) ... 100 (100)

---

Q7 Does your unit support adult or paediatric/neonatal patients, or both?

◯ Adult (1)

◯ Paediatric/Neonatal (2)

◯ Both (3)

---

Q8 Has your unit had any patients with COVID-19?

◯ Yes (1)

◯ No (2)

---

*Display This Question:*

*If Has your unit had any patients with COVID-19? = No*

Q9 Has your unit been referred any patients that have COVID-19, but were subsequently declined by your unit?

◯ Yes (1)

◯ No (2)

---

Q10 In a usual month (prior to the pandemic), what proportion of your unit's maximum available beds for ECMO are in use?

- (1)
- 20-50% (2)
- 50-80% (3)
- >80% (4)

---

Q11
In the past month (during the pandemic), what proportion of your unit's maximum available beds for ECMO have been in use?

- (1)
- 20-50% (2)
- 50-80% (3)
- >80% (4)

**End of Block: Unit**

---

**Start of Block: Decisions about ECMO - ethics**

Q12 Please indicate on the given scale the extent to which you agree or disagree with the following statements.

| | Strongly disagree (1) | Disagree (2) | Somewhat disagree (3) | Neutral (4) | Somewhat agree (5) | Agree (6) | Strongly agree (7) |
|---|---|---|---|---|---|---|---|
| When thinking about how decisions are made about ECMO in your unit, the most important thing is to give every patient an equal chance of treatment, even if that means doing less good overall. (1) | ○ | ○ | ○ | ○ | ○ | ○ | ○ |
| When thinking about how decisions are made about ECMO in your unit, the most important thing is to do the most good overall, even if that means | ○ | ○ | ○ | ○ | ○ | ○ | ○ |

| |
|---|
| not giving every patient an equal chance. (2) |

Q13 In usual times (prior to the pandemic), when making decisions about starting ECMO, how much do you or your team consider the benefit to the individual being referred versus the potential benefit of ECMO to other patients?

| | We consider only the benefit to this patient | We consider only which patient would benefit most |
|---|---|---|
| | 0 10 20 30 40 50 | 60 70 80 90 100 |
| Please indicate your response on the given scale. () | | |

Q47 Since the start of the pandemic, when making decisions about starting ECMO, how much have you or your team considered the benefit to the individual being referred versus the potential benefit of ECMO to other patients?

| | We consider only the benefit to this patient | We consider only which patient would benefit most |
|---|---|---|
| | 0 10 20 30 40 50 | 60 70 80 90 100 |
| Please indicate your response on the given scale. () | | |

End of Block: Decisions about ECMO - ethics

Start of Block: Decisions starting ECMO - usual

Q14 In usual circumstances (prior to the pandemic), how often would each of the following factors below be included in decisions about starting ECMO in your unit?

| | Always (1) | Often (2) | Sometimes (3) | Never (4) | Has not arisen but would consider it (5) |
|---|---|---|---|---|---|
| Probability of survival if treated with ECMO (1) | ○ | ○ | ○ | ○ | ○ |
| Expected duration on ECMO (2) | ○ | ○ | ○ | ○ | ○ |
| Predicted length of survival post ECMO (3) | ○ | ○ | ○ | ○ | ○ |
| Predicted quality of survival post ECMO (4) | ○ | ○ | ○ | ○ | ○ |
| Pre-existing disability (5) | ○ | ○ | ○ | ○ | ○ |
| Functional status (6) | ○ | ○ | ○ | ○ | ○ |
| Age (7) | ○ | ○ | ○ | ○ | ○ |
| The patient is a Health Care Worker (8) | ○ | ○ | ○ | ○ | ○ |
| The patient has dependents (9) | ○ | ○ | ○ | ○ | ○ |
| Positive social value (e.g. scientist, police) (10) | ○ | ○ | ○ | ○ | ○ |
| Negative social value (e.g. violent criminal) (11) | ○ | ○ | ○ | ○ | ○ |

| | | | | | |
|---|---|---|---|---|---|
| The number of ECMO beds already occupied (12) | ○ | ○ | ○ | ○ | ○ |

*Display This Question:*

*If In usual circumstances (prior to the pandemic), how often would each of the following factors bel... != Probability of survival if treated with ECMO [ Never ]*

Q15 What probability of survival would be too low to start ECMO? I.e. if a patient were predicted to have this chance or lower of survival (with ECMO) you would not start ECMO?

| | 0 10 20 30 40 50 60 70 80 90 100 |
|---|---|
| Probability of survival (%) () | |

*Display This Question:*

*If In usual circumstances (prior to the pandemic), how often would each of the following factors bel... != Age [ Never ]*

Q16 You have indicated that age might be included in decisions regarding starting ECMO. In usual circumstances (prior to the pandemic), do you have an upper age limit for ECMO? If so, what is it?

○ Yes (1) ________________________________________________

○ No (2)

*Display This Question:*

*If In usual circumstances (prior to the pandemic), how often would each of the following factors bel... != Age [ Never ]*

Q17 In thinking about why age is included in decisions about ECMO, please indicate on the given scale the extent to which you agree or disagree with the below statements.

| | Strongly disagree (1) | Disagree (2) | Somewhat disagree (3) | Neutral (4) | Somewhat agree (5) | Agree (6) | Strongly agree (7) |
|---|---|---|---|---|---|---|---|
| Age is relevant because older patients would not survive even if they were treated with ECMO. (1) | o | o | o | o | o | o | o |
| Age is relevant because age predicts the chance of the patient surviving. (2) | o | o | o | o | o | o | o |
| It would be better to treat a younger patient rather than an older patient with ECMO even if they had identical chance of survival (3) | o | o | o | o | o | o | o |
| Age is relevant because younger patients will potentially survive for longer (ie more years) | o | o | o | o | o | o | o |

| | | | | | | | |
|---|---|---|---|---|---|---|---|
| after ECMO. (4) | | | | | | | |
| Age is relevant because older patients are less likely to have dependents. (5) | o | o | o | o | o | o | o |
| Age is relevant because older patients have already lived longer. (6) | o | o | o | o | o | o | o |

End of Block: Decisions starting ECMO - usual

Start of Block: Decisions starting ECMO - pandemic

Q18 Since the start of the pandemic, how often would each of the following factors below be included in decisions about starting ECMO in your unit?

| | Always (1) | Often (2) | Sometimes (3) | Never (4) | Has not arisen but would consider it (5) |
|---|---|---|---|---|---|
| Probability of survival if treated with ECMO (1) | ○ | ○ | ○ | ○ | ○ |
| Expected duration on ECMO (2) | ○ | ○ | ○ | ○ | ○ |
| Predicted length of survival post ECMO (3) | ○ | ○ | ○ | ○ | ○ |
| Predicted quality of survival post ECMO (4) | ○ | ○ | ○ | ○ | ○ |
| Pre-existing disability (5) | ○ | ○ | ○ | ○ | ○ |
| Functional status (6) | ○ | ○ | ○ | ○ | ○ |
| Age (7) | ○ | ○ | ○ | ○ | ○ |
| The patient is a Health Care Worker (8) | ○ | ○ | ○ | ○ | ○ |
| The patient has dependents (9) | ○ | ○ | ○ | ○ | ○ |
| Positive social value (e.g. scientist, police) (10) | ○ | ○ | ○ | ○ | ○ |
| Negative social value (e.g. violent criminal) (11) | ○ | ○ | ○ | ○ | ○ |

*Display This Question:*

*If Since the start of the pandemic, how often would each of the following factors below be included... != Age [ Never ]*

Q19 You have indicated that age might be included in decisions regarding starting ECMO. In current circumstances (during the pandemic), do you have an upper age limit for ECMO? If so, what is it?

- ◯ Yes (1) ________________________________________________
- ◯ No (2)

*Display This Question:*

*If Since the start of the pandemic, how often would each of the following factors below be included... != The patient is a Health Care Worker [ Never ]*

Q20 Please indicate whether you agree or disagree with the following statement.

If would be ethical to give extra priority for ECMO to a healthcare worker who had contracted COVID-19 whilst working.

- ◯ Agree (1)
- ◯ Disagree (2)

*Display This Question:*

*If Since the start of the pandemic, how often would each of the following factors below be included... != The patient is a Health Care Worker [ Never ]*

Q21 Would you consider treating a healthcare worker with COVID-19 above your usual age limit?

- ◯ Yes (1)
- ◯ No (2)

Q22 Have your criteria for starting ECMO changed during the COVID-19 pandemic? If so, in what way?

- ○ Yes (1) ________________________________________________
- ○ No (2)

Q33 Have your number of available beds for ECMO changed during the COVID-19 pandemic? If so, in what way?

- ○ Yes (1) ________________________________________________
- ○ No (2)

End of Block: Decisions starting ECMO - pandemic

Start of Block: Decisions stopping ECMO

Q23 In decisions about stopping ECMO, how much do you or your team consider the benefit to the individual patient currently being treated versus the potential benefit of ECMO to other patients?

| | We consider only the benefit to this patient | We consider only which patient would benefit most |
|---|---|---|
| | 0 10 20 30 40 50 | 60 70 80 90 100 |
| Please indicate your response on the given scale. () | [slider] | |

Q24 What probability of survival would be too low to continue ECMO? I.e. if a patient who is already on ECMO were predicted to have this chance or lower of survival you would not continue ECMO

0 10 20 30 40 50 60 70 80 90 100

| | |
|---|---|
| Probability of survival (%) () | |

Q25 In usual circumstances (prior to the pandemic), how many days of ECMO would you consider reasonable before recommending that it stop?

▼ 1 (1) ... 1000 (1000)

Q26 In usual circumstances, if all of the ECMO circuits/machines were in use and a good candidate patient were referred for ECMO, would you consider discontinuing ECMO for one of the current patients (who has a low, but non-zero chance of survival) in order to provide ECMO to the referred patient?

- Yes (1)
- No (2)

Q27 In usual circumstances, if your clinical team recommends that ECMO should cease, but the family do not agree, do you continue treatment? If so, for how many days?

- Yes (1) ________________________________________________
- No (2)

Q28 During the pandemic, how many days of ECMO would you consider reasonable before recommending that it stop?

▼ 1 (1) ... 1000 (1000)

Q29 During the pandemic, if all of the ECMO circuits/machines were in use and a good candidate patient were referred for ECMO, would you consider discontinuing ECMO for one of

the current patients (who has a low, but non-zero chance of survival) in order to provide ECMO to the referred patient?

- Yes (1)
- No (2)

---

Q30 During the pandemic, if your clinical team recommends that ECMO should cease, but the family do not agree, do you continue treatment? If so, for how many days?

- Yes (1) ________________________________________________
- No (2)

End of Block: Decisions stopping ECMO

---
